# A correction for modeling radial, spiral, and PROPELLER dynamic contrast‐enhanced data: Time‐averaged extended Tofts

**DOI:** 10.1002/mrm.30514

**Published:** 2025-03-30

**Authors:** Natalia V. Korobova, Nienke P. M. Wassenaar, Marian A. Troelstra, Eric M. Schrauben, Oliver J. Gurney‐Champion

**Affiliations:** ^1^ Department of Radiology and Nuclear Medicine Amsterdam University Medical Center Amsterdam The Netherlands; ^2^ Imaging and Biomarkers Cancer Center Amsterdam Amsterdam The Netherlands

**Keywords:** dynamic contrast‐enhanced MRI, extended Tofts model, k‐space sampling patterns, pancreatic cancer, perfusion

## Abstract

**Purpose:**

Dynamic contrast‐enhanced sequences (e.g. spiral, radial, PROPELLER MRI) often rely on oversampling the center of k‐space. Instead of the discrete snapshots obtained by Cartesian sampling, oversampling the k‐space center results in time‐averaging of the signal. We hypothesize that these time‐averaged signals decrease the accuracy of pharmacokinetic modeling and propose a model that accounts for this effect.

**Theory and Methods:**

To test our hypothesis, a modified extended Tofts model tailored to accommodate time‐averaged signals is proposed. Simulated Monte Carlo experiments were conducted to compare the performance of the modified model with the conventional model. Additionally, to validate the findings in vivo, models were fitted to pseudo‐spiral variable‐density dynamic contrast‐enhanced MRI scans of pancreatic cancer patients reconstructed at 4, 8, 10, and 15 s/frame.

**Results:**

The simulations demonstrated that for time‐averaged acquisitions, our modified extended Tofts model provided more accurate and precise results than conventional models. Additionally, by integrating signals, some information on high temporal behavior was recovered. Particularly, at long acquisitions (15 s/frame), variable‐density sampling with the modified model outperformed conventional discrete sampling. In vivo experiments confirmed these findings, as the corrected model showed more consistent estimates of parameters $v_{p}$ and $v_{e}$ over the tested sampling frequencies, highlighting its potential to improve accuracy in clinical settings.

**Conclusion:**

Our study demonstrates that time‐averaged signals lead to decreased accuracy and precision in pharmacokinetic modeling when ignored. We suggest using our corrected pharmacokinetic model when performing dynamic contrast‐enhanced with variable‐density acquisitions, especially for dynamic scan times that are 8 s and longer.

## 1 INTRODUCTION

Dynamic contrast‐enhanced (DCE) MRI offers crucial pathophysiological insight across various areas of clinical research, such as oncology, neurovascular disease, and neurodegenerative conditions.^1^, ^2^, ^3^, ^4^, ^5^ DCE‐MRI is performed by a dynamic acquisition of $T_{1}$‐weighted images before and after the intravenous administration of exogenous gadolinium‐based contrast agent. The paramagnetic properties of the contrast agent shorten local $T_{1}$ relaxation time in blood and tissues; hence, a physiology‐dependent signal increase occurs in $T_{1}$‐weighted images. Through pharmacokinetic modeling, physiological parameters describing tissue microvascular perfusion and vascular permeability can be determined from the DCE signaltime curve.^6^

**FIGURE 1:**
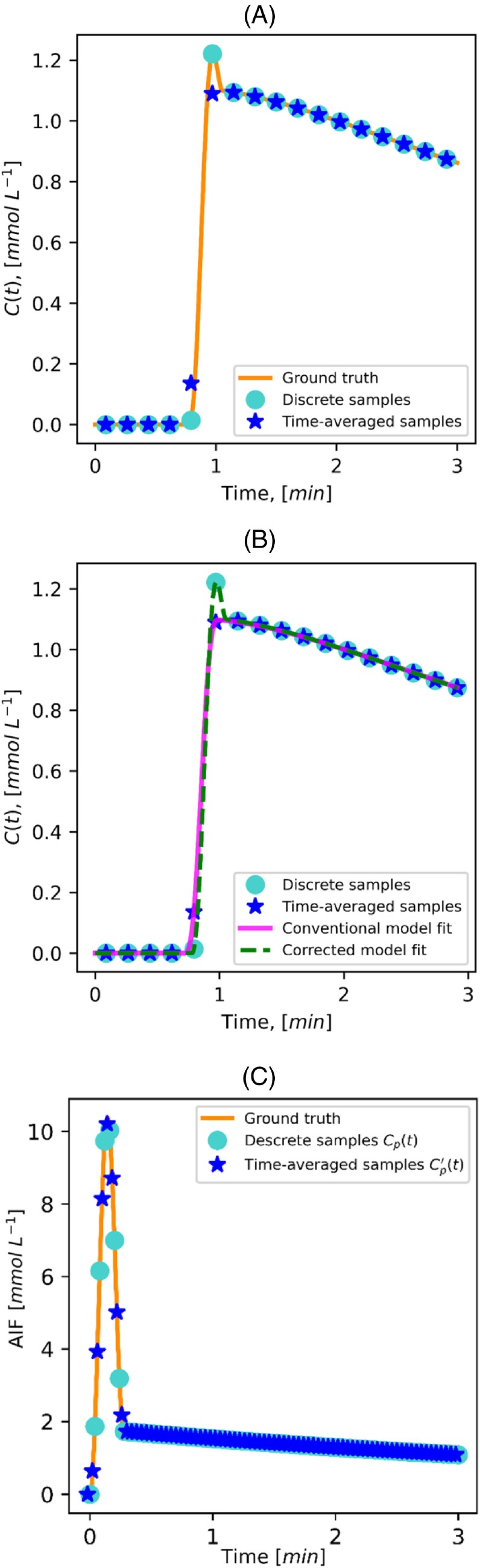
(A) Example contrastconcentration curve showing the ground‐truth curve (*orange line*), discrete sampling points using a conventional Cartesian acquisition (*light blue circles*), and time‐averaged sampling points obtained with a variable‐density trajectory that oversamples the center of k‐space (*dark blue stars*). (B) Example of fitting conventional (*pink line*) and corrected (*green line*) pharmacokinetic models to time‐averaged samples, highlighting the differences in model accuracy. (C) Example arterial input function (AIF) curve with its discrete sampling (*light blue circles*) and time‐averaged sampling (*dark blue stars*) shown for comparison.

Conventional pharmacokinetic models, including the extended Tofts model,^7^, ^8^ describe the contrast agent concentration (C) as a continuous function of time (t): C(t). These concentration time curves are typically derived from signal time curves using (1) an equation relating changes in $T_{1}$ values to changes in the signal^9^ and (2) an equation relating changes in $T_{1}$ to contrast concentration. Pharmacokinetic parameters are then fitted to discrete concentration samples at time points corresponding to individual dynamic scans in the DCE series. This approach perfectly reflects the conventional Cartesian sampling, in which the center of k‐space, which predominantly determines the image contrast, is sampled once per frame (Figure 1A).

Accurate quantitative modeling of the DCE signal requires a high temporal resolution,^10^ which is difficult to achieve over a three‐dimensional (3D) volume (e.g., whole liver) using conventional Cartesian sampling. Recently, high‐temporal‐resolution volumetric DCE has been achieved using variable density sampling patterns, including radial,^11^ (pseudo‐) spiral,^12^, ^13^ and PROPELLER^14^ techniques. In these acquisitions, the center of k‐space is acquired multiple times during each DCE frame. Consequently, the signal is averaged over the acquisition time for each temporal frame (Figure 1A). Pharmacokinetic models do not take this into account and therefore may give biased parameter estimates (Figure 1B). Hence, these acquisition techniques for DCE modeling need to incorporate time‐averaging behavior in signal modeling.

Moreover, such time‐averaged signals inherently contain some information about faster processes that occur within their temporal frame, which would have been missed by conventional discrete snapshot acquisitions. For example, the peak of the contrast‐agent bolus could be completely missed by the discrete sampling but will influence a time‐averaged signal. Time‐averaged acquisitions may therefore potentially retain information about fast processes even at slow scan times. This is particularly relevant for the supplying artery, where the concentration time curve of the contrast agent, known as the arterial input function (AIF), is crucial for accurate pharmacokinetic modeling^15^ (Figure 1C).

We hypothesize that these time‐averaged signals decrease the accuracy of conventional pharmacokinetic modeling. We further hypothesize that they can result in more accurate parameter estimates when properly modeled. Here we introduce a modified extended Tofts model tailored to account for time‐averaged signals. We then use Monte Carlo experiments to explore to what extent time‐averaged signals influence DCE modeling when ignored, and whether this can be improved by our new corrected model. Finally, we demonstrate its performance in vivo in pancreatic cancer patients.

### 1.1 Theory

#### 1.1.1 DCE model

In this work, the extended Tofts model^7^, ^8^ is used to quantify pharmacokinetic tissue properties. This model assumes that the contrast agent diffuses between two compartments: blood plasma and extracellular‐extravascular space. The concentration of the contrast agent at every time point, t, is expressed through pharmacokinetic parameters, $u=\left[v_{p},v_{e},K^{\text{trans}}\right]$, as follows:

(1) $$\begin{array}{cc} C\left(t,u,C_{p}\left(t\right)\right) & =v_{p}C_{p}\left(t\right)+K^{\text{trans}}C_{p}\left(t\right)*e^{-\frac{K^{\text{trans}}}{v_{e}}\;t}=v_{p}C_{p}\left(t\right)\\ & \;+K^{\text{trans}}\int_{0}^{t}C_{p}\left(\tau \right)e^{-\frac{K^{\text{trans}}}{v_{e}}\left(t-\tau \right)}d\tau , \end{array}$$

where $v_{p}$ [fraction] is the volume of plasma; $v_{e}$ [fraction] is the volume of extracellular‐extravascular space; $\;K^{\text{trans}}$ [min^−1^] is the influx mass transfer rate; $C_{p}$ [mmolL^‐1^] is the concentration of the contrast agent in the blood plasma, expressed as the AIF; and * represents a convolution operation. The reflux rate of contrast $k_{\mathrm{ep}}$ [min^−1^] is defined as the ratio between $K^{\text{trans}}$ over $v_{e}$.

#### 1.1.2 Modification of the model

To describe the time‐averaged signal per acquisition, we propose to modify the extended Tofts model. To achieve signal averaging over time, we analytically convolve the pharmacokinetic model with a rectangular function with a *rect*‐width equivalent to the acquisition time of a DCE frame, ∆t, as follows:

(2) $$\begin{array}{cc} C^{\prime }\left(t,u,C_{p}\right) & =\frac{1}{∆t}C\left(t,u,C_{p}\right)*\text{rect}\left(\frac{t}{∆t}\right)\\ & \;=\frac{1}{∆t}\int_{t-\frac{1}{2}∆t}^{t+\frac{1}{2}∆t}C\left(\tau ,u,C_{p}\right)d\tau , \end{array}$$

where $C^{\prime }\left(t,u,C_{p}\right)$ represents the time‐averaged extended Tofts model. Although $C\left(t,u,C_{p}\right)$ is defined as the extended Tofts model in Eq. (1) for this work, in principle, Eq. (2) is general and holds for all pharmacokinetic models. Mathematically, the convolution of the extended Tofts model (Eq. [1]) with the rectangular function as described in Eq. (2) is equivalent to only convolving the AIF with the following rectangular function:

(3) $$\begin{array}{c} C^{\prime }\left(t,u,C_{p}\right)=C\left(t,u,{C_{p}}^{\prime }\left(t\right)\right), \end{array}$$

with

(4) $$\begin{array}{c} C_{p}^{\prime }\left(t\right)=\frac{1}{∆t}C_{p}\left(t\right)*\text{rect}\left(\frac{t}{∆t}\right), \end{array}$$

where $C_{p}^{\prime }\left(t\right)$ represents the time‐averaged AIF. The proof is provided in Supporting Information S1. Hence, the convolution of the AIF with the rectangular function is theoretically sufficient for the correction; measuring the AIF with the same time‐averaged acquisition as the DCE scan (e.g., patient‐specific AIF), and using conventional pharmacokinetic modeling (Eq. [1]), theoretically should not result in a bias.

## 2 METHODS

All analyses were done in *Python* (v3.9.16; Python Software Foundation). We used the extended Tofts model implementation and fit routines from the *Python* implementation from OG_MO_AUMC_ICR_RMH_NL_UK^16^ from OSIPI GitHub^17^ as standard DCE Equation (1). The chosen model contains an analytical representation of AIF, $C_{p}\left(t,\theta_{\mathrm{AIF}}\right)$, dependent on a set of four parameters $\theta_{\mathrm{AIF}}$, that describes the curve as an initial pass, followed by wash‐out (defined in Supporting Information S2). Parameters $\theta_{\mathrm{AIF}}$ define the shape of AIF. This analytical form of AIF simplifies the mathematical integration in Eq. (1) with an analytical solution.

To implement the time‐averaged variant (Eq. [2]), we analytically convolved this implementation with a block function, for which the solution can be found in Supporting Information S3 and on the OSIPI repository: https://github.com/OSIPI/DCE‐DSC‐MRI_CodeCollection/tree/develop/src/original/NK_OG_MO_AUMC_ICR_RMH_NL_UK.

All models were fitted with the nonlinear least squares algorithm with the following constraints: $v_{e}$ [0–1], $k_{\mathrm{ep}}$ [0–3], and $v_{p}$ [0–1].

We first address our hypothesis in a controlled simulated setting. Subsequently, we show the performance in vivo.

### 2.1 Simulations

#### 2.1.1 Data

**FIGURE 2:**
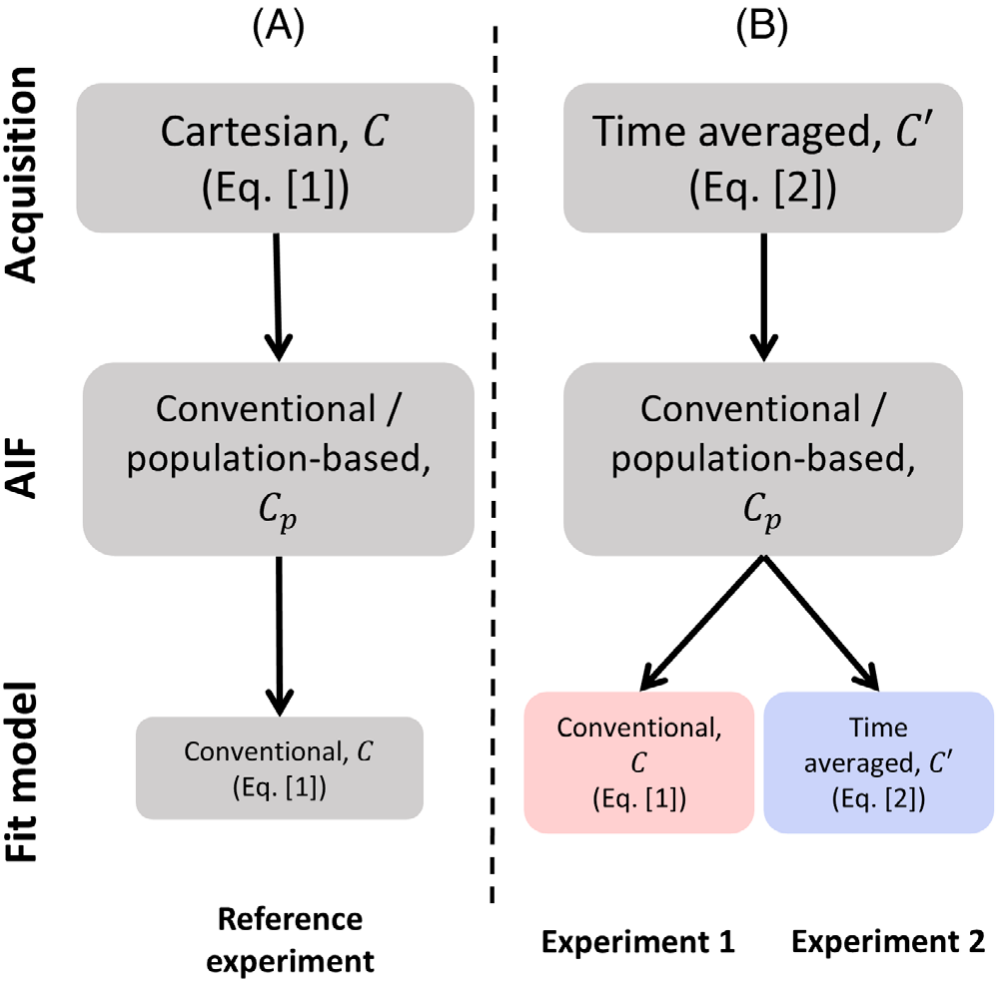
Overview of experiments conducted in simulations. (A) Reference experiment replicated temporally discrete data acquired with Cartesian trajectory. The conventional extended Tofts model was used in the fitting of pharmacokinetic parameters. (B) Alternative experiments simulated temporally averaged concentration curves resembling acquisition trajectories that oversample the center of k‐space. Both conventional and corrected extended Tofts models were used for fitting. A population‐based AIF was used across all experiments.

To evaluate the effect of signal averaging on the pharmacokinetic modeling in a controlled environment, we performed several Monte Carlo simulations. Each simulation contained 50 000 contrast‐agent concentration curves $C\left(t,u,\theta_{\mathrm{AIF}}\right)$ with a broad range of different pharmacokinetic parameters. The parameters $v_{e}$ [0–1] and $k_{\mathrm{ep}}$ [0–3] were sampled from a uniform distribution and $v_{p}$ from an exponential distribution with a rate parameter λ=40, corresponding to a mean of 0.025, within the range [0–0.37]. To examine the performance over the entire range of parameter $v_{p}$, we simulated a similar data set, in which $v_{p}$ was sampled uniformly [0–1]. Boundaries of simulated parameters included typically observed values in the pancreas, muscle, spleen, and liver.^18^ Random Gaussian noise was added to the curves. The standard deviation of the noise was the same for both $C\left(t,u,\theta_{\mathrm{AIF}}\right)$ and $C^{\prime }\left(t,u,\theta_{\mathrm{AIF}}\right)$. For a given curve, the standard deviation of noise was constant, meaning that signal‐to‐noise ratio (SNR) changed over time. The standard deviation of the noise was chosen such that the ratio of the mean signal in the time‐averaged curve $C^{\prime }\left(t,u,\theta_{\mathrm{AIF}}\right)$ divided by this standard deviation gave an SNR of 20. We simulated 5 min of $C\left(t,u,\theta_{\mathrm{AIF}}\right)$ at various temporal resolutions ∆t: 4, 8, 10, and 15 s/frame. Simulated signal curves were sampled using both of the following (Figure 2):

- A temporally discrete Cartesian acquisition $C\left(t,u,\theta_{\mathrm{AIF}}\right)$ (Eq. [1])
- A temporally averaged variable density acquisition $C^{\prime }\left(t,u,\theta_{\mathrm{AIF}}\right)$ (Eq. [2])

#### 2.1.2 Experiments

First, we answered our first hypothesis and investigated how time‐averaged signals decreased the accuracy of conventional pharmacokinetic modeling. We fitted the conventional extended Tofts model to (A) temporally averaged signal (Figure 2: Experiment 1) and, as a reference, to (B) discrete signal (Figure 2: Reference experiment).

Then, we answered our second hypothesis and investigated whether using our corrected equation allows for correct estimates. This was achieved using our proposed fit (Figure 2: Experiment 2) and comparing it with Experiment 1. To investigate whether time averaging indeed better retains pharmacokinetic information, Experiment 2 was also compared with the reference experiment. For all experiments, $\theta_{\mathrm{AIF}}$ was assumed to be known, reflecting a population‐based AIF (e.g., from literature,^19^ obtained with discrete acquisitions reflecting ground‐truth curves). Parameters $\theta_{\mathrm{AIF}}$ were then incorporated into analytically expressed AIF, which further was used in both conventional and corrected extended Tofts models.

Additionally, we extended Experiments 1 and 2 by changing a population‐based AIF with a convolved AIF $C_{p}^{\prime }$ (with the equations from Supporting Information S3), reflecting the measurement of a patient‐specific AIF with a variable density acquisition. The results are not included in the main text but are added to Supporting Information S4.

#### 2.1.3 Evaluation

The performance of models was estimated in terms of accuracy (measured by the mean error in the fitted pharmacokinetic parameters) and precision (represented by the standard deviation of these errors). Differences in accuracy across various experiments were tested with a paired Wilcoxon signed‐rank test, whereas the difference in precision was assessed with a paired Levene test. A *p*‐value below 0.05 was considered significant.

### 2.2 In vivo

We validated our methods in vivo, where no ground‐truth reference is available. Instead, we assessed whether our proposed method keeps pharmacokinetic parameters consistent across different reconstructed dynamic times. In contrast, the conventional approach may introduce bias when the time per dynamic is too long. To test this, we reconstructed the data at 4, 8, 10, and 15 s per frame.

#### 2.2.1 Data acquisition

Eight patients diagnosed with pancreatic ductal adenocarcinoma underwent an abdominal DCE‐MRI examination. The study was approved by the ethics committee of the Amsterdam UMC location AMC (NL73810.018.20), and all participants gave written consent before the start of the study. MRI scans were performed on a 3T system (Ingenia; Philips, Best, The Netherlands), using a 16‐channel phased‐array anterior coil and a 16‐channel phased‐array posterior receive coil. After 30 s, 0.2 mL/kg of 0.5 mmol/mL gadoterate meglumine (Dotarem; Guerbet, Villepinte, France) followed by a 15 mL saline flush were administered at a rate of 5 mL/s. The DCE protocol consisted of a dynamic series of a 3D pseudo‐spiral turbo field echo–spoiled gradient‐echo sequence with spectral‐attenuated inversion‐recovery fat suppresion.^13^ Pseudo‐spiral sampling was achieved using a modified version of the free‐running Amsterdam UMC (“Prospective Undersampling in Multiple Dimensions” [PROUD]) software patch (https://mriresearch.amsterdam/software/aumcproudpatch/).^20^, ^21^ The imaging parameters were as follows: 3D axial (right–left readout) orientation with field of view = 374 × 258 × 105 mm, flip angle = 25°, turbo field echo shot length = 50 readouts, spectral‐attenuated inversion‐recovery prepulse duration = 61.1 ms, repetition time/echo time = 3.8/1.8 ms, number of slices = 35, matrix size = 220 × 152, slice thickness = 3 mm, in‐plane resolution = 1.7 mm, and acquisition time = 7 min. The dynamic series were reconstructed in *MATLAB* (R2023a; MathWorks) with the BART Toolbox (v0.5.00; https://mrirecon.github.io/bart/) using compressed sensing^22^, ^23^ with regularization in the temporal (total variation *λ* = 0.05) and spatial (total variation *λ* = 0.05) domain at temporal resolutions of 4, 8, 10, and 15 s/frame. Participants were instructed to breathe freely during the entire DCE image acquisition. Post hoc breathing motion correction was performed based on an auto‐focus approach^24^ as described by Wassenaar et al.^13^

#### 2.2.2 Data postprocessing

Before pharmacokinetic modeling, the dynamic signal was converted to the concentration of the contrast agent with the spoiled gradient‐echo signal model. The baseline $T_{1}$ time was set to 725 ms for the pancreas.^25^

Similar to the simulations, pharmacokinetic parameters were calculated using the conventional (Eq. [1]) and corrected (Eq. [2]) models. A population‐based AIF was used and was adjusted for an average hematocrit fraction of a healthy adult of 42%.^26^

#### 2.2.3 Evaluation

For all 8 patients, the pharmacokinetic parameters were calculated within a manually segmented region of interest (ROI) in the tumor. Additionally, these parameters were measured in non‐tumorous pancreatic tissue in 6 patients—2 patients were excluded due to atrophy of the pancreas. Delineation of ROIs was performed by a researcher with 4 years of experience in pancreatic imaging on the DCE‐MRI scan with a 15‐s temporal resolution. The arterial phase frame (between 20 s and 35 s following contrast injection) was manually selected for ROI placement. The clinically performed contrast‐enhanced CT scan and a $T_{2}$‐weighted turbo spin‐echo MRI scan made during the research MR protocol were used for anatomical guidance. The performance of the models was considered to be robust when predictions of pharmacokinetic parameters were consistent across different temporal resolutions. Therefore, to estimate the accuracy of both models, we calculated the mean absolute difference between predictions derived from the fastest dynamic series (4 s/frame) and the remaining series for each subject. We used a paired signed‐rank Wilcoxon test to assess whether both methods significantly differ.

## 3 RESULTS

### 3.1 Simulations

**FIGURE 3:**
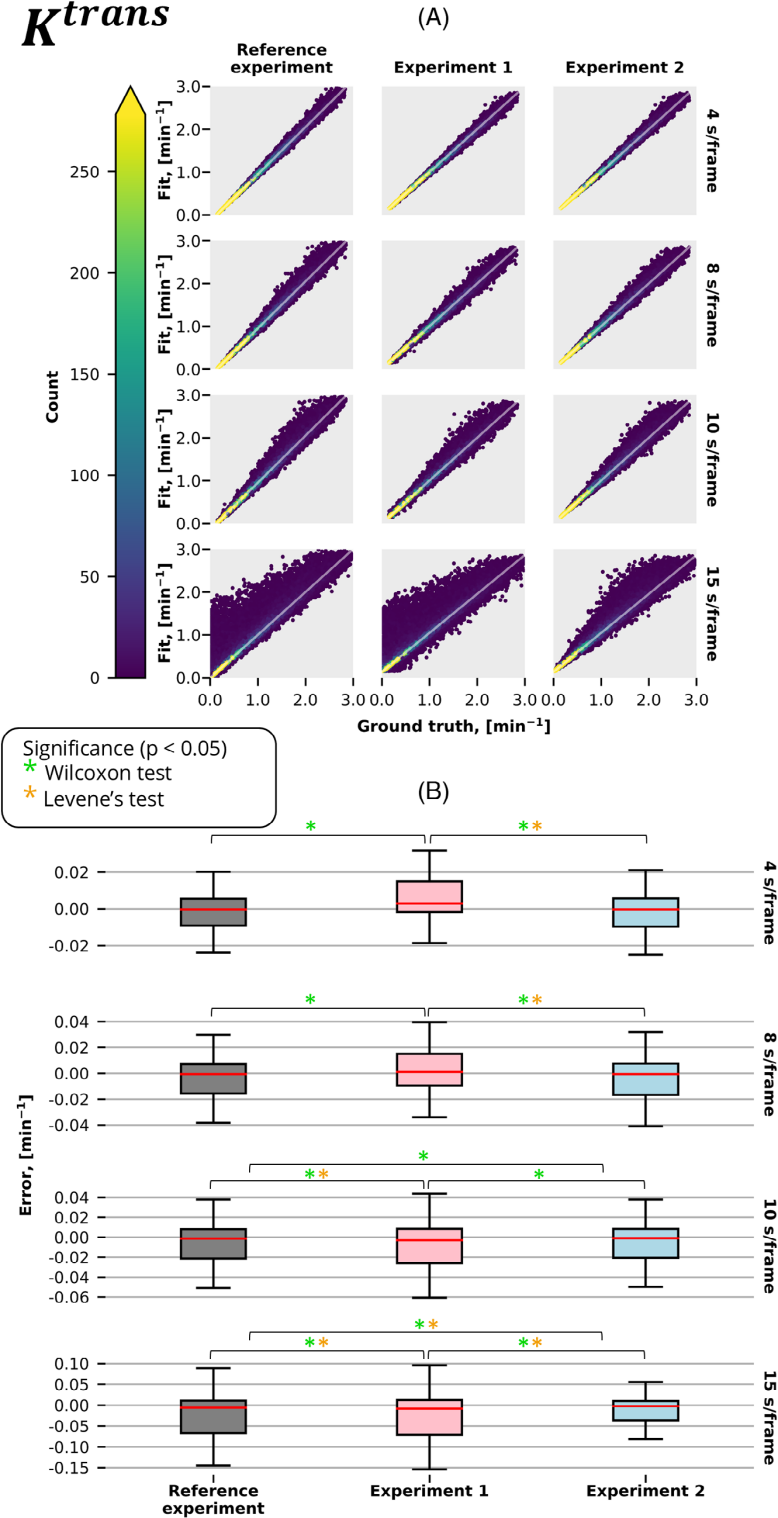
The $K^{\text{trans}}$ results of the experiments described in Figure 2 were conducted at various temporal resolutions. (A) The graph displays the fitted value of the parameter $K^{\text{trans}}$ plotted against the ground truth. The color gradient represents the density of points, whereas the white diagonal line represents the function Fit = Ground Truth. (B) Distribution of errors in the predicted parameter $K^{\text{trans}}$ for the same experiments and temporal resolutions. Significant differences, as identified by the Wilcoxon (accuracy) and Lavene (precision) tests (*p* < 0.05), are denoted by green and orange stars, respectively.

**FIGURE 4:**
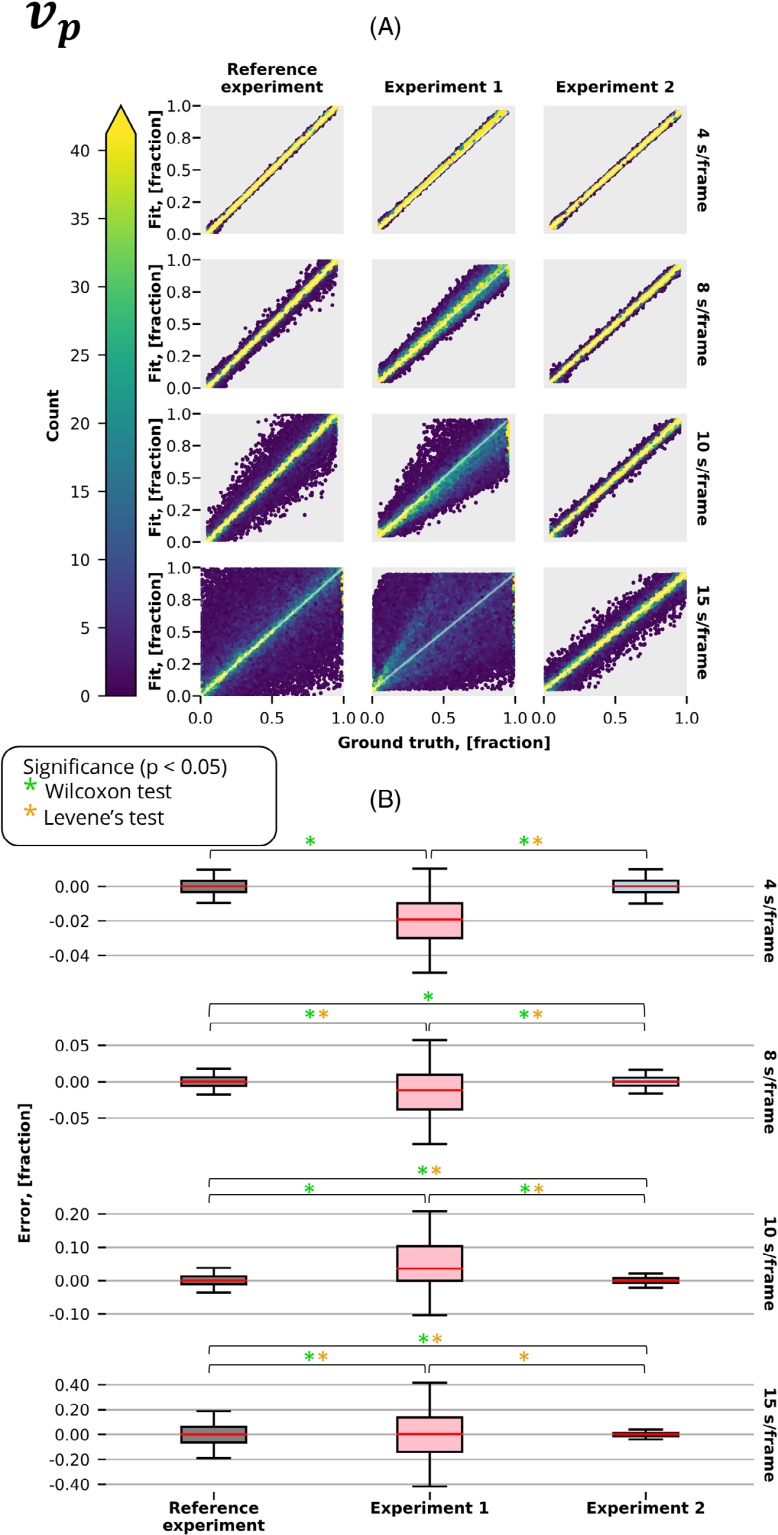
The $v_{p}$ results of the experiments described in Figure 2 were conducted at various temporal resolutions. (A) Graph displays the fitted value of the parameter $v_{p}$ plotted against the ground truth. The color gradient represents the density of points at each location, whereas the white diagonal line represents the function Fit = Ground Truth. (B) Distribution of errors in the predicted parameter $v_{p}$ for the same experiments and temporal resolutions. Significant differences, as identified by the Wilcoxon (accuracy) and Lavene (precision) tests (*p* < 0.05), are denoted by green and orange stars, respectively.

**FIGURE 5:**
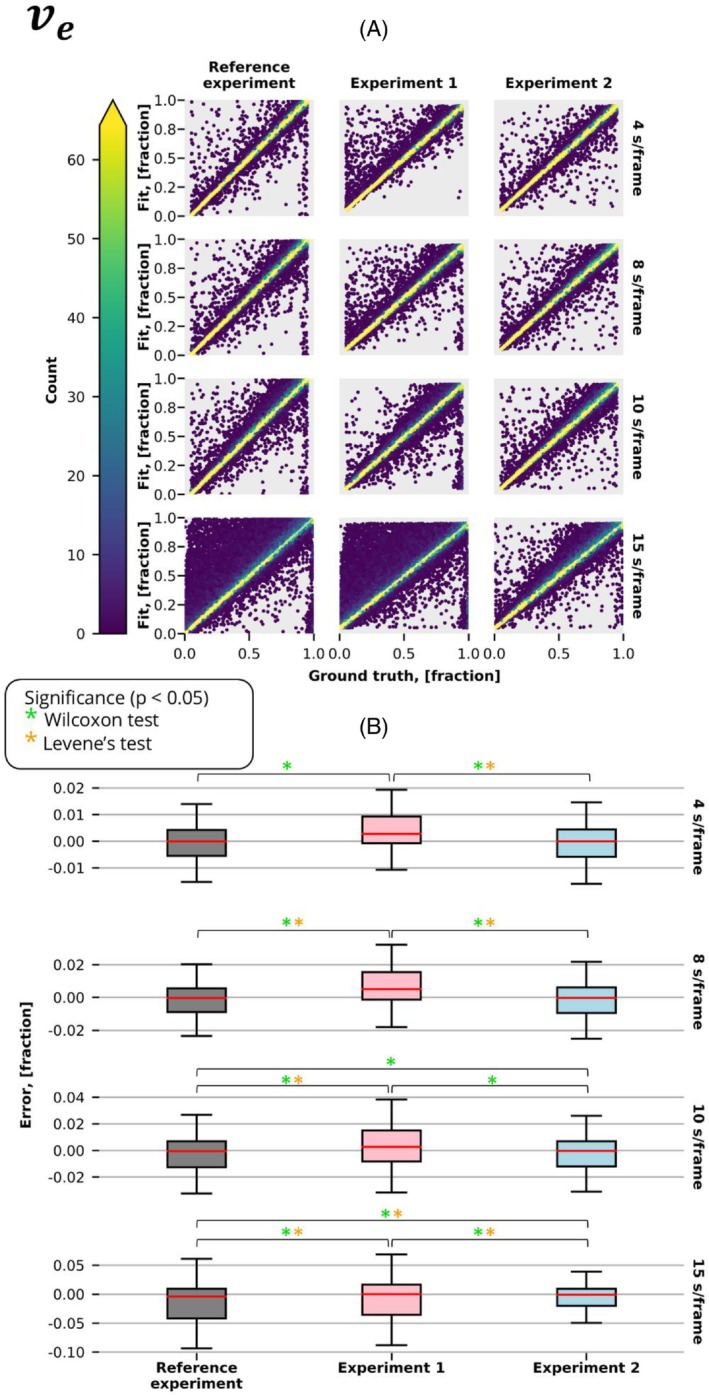
The $v_{e}$ results of the experiments described in Figure 2 were conducted at various temporal resolutions. (A) Graph displays the fitted value of the parameter $v_{e}$ plotted against the ground truth. The color gradient represents the density of points at each location, whereas the white diagonal line represents the function Fit = Ground Truth. (B) Distribution of errors in the predicted parameter $v_{e}$ for the same experiments and temporal resolutions. Significant differences, as identified by the Wilcoxon (accuracy) and Lavene (precision) tests (*p* < 0.05), are denoted by green and orange stars, respectively.

Figures 3, 4, and 5 present estimated parameters $K^{\text{trans}},v_{p},v_{e}$ obtained from fitting the extended Tofts model with and without correction for discretely sampled (Figure 2A) and time‐averaged (Figure 2B) signals acquired at different temporal resolutions. As anticipated, all experiments showed superior performance at the highest temporal resolution, where averaging is almost identical to discrete sampling, and decreased performance at lower temporal resolutions.

When averaging is not accounted for, measuring with a time‐averaged signal (Experiment 1) had a substantial decrease in performance compared with conventional Cartesian measurements (reference experiment). Particularly, we observed significantly better precision in 7 of 12 comparisons (3 parameters, 4 temporal resolutions) and accuracy in 12 of 12 comparisons.

When measuring a time‐averaged signal, the correction in pharmacokinetic modeling (Experiment 2) produced significantly more accurate (12 of 12 comparisons) and precise (9 of 12 comparisons) estimates in comparison to the conventional model.

Interestingly, at longer temporal resolutions (10 and 15 s/frame), the conventional Cartesian measurements resulted in substantially poorer estimates when compared with the time‐averaged model. Particularly, accuracy was significantly improved in 4 of 6 comparisons (3 parameters, 2 temporal resolutions) and precision in 6 of 6.

### 3.2 In vivo

**FIGURE 6:**
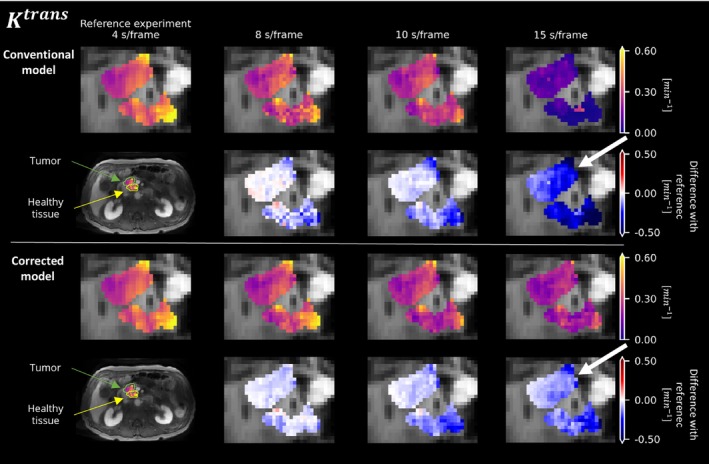
Estimation of the parameter $K^{\text{trans}}$ in the tumor (*green arrow*) and healthy tissue (*yellow arrow*) of a pancreatic ductal adenocarcinoma patient. The results were calculated using both the conventional model (*Row 1*) and the corrected model (*Row 3*) at different temporal resolutions. Additionally, the corresponding differences in predictions (Rows 2 and 4) across various temporal resolutions compared with the reference (Column 1), which correspond to the fastest acquisition, are shown.

**FIGURE 7:**
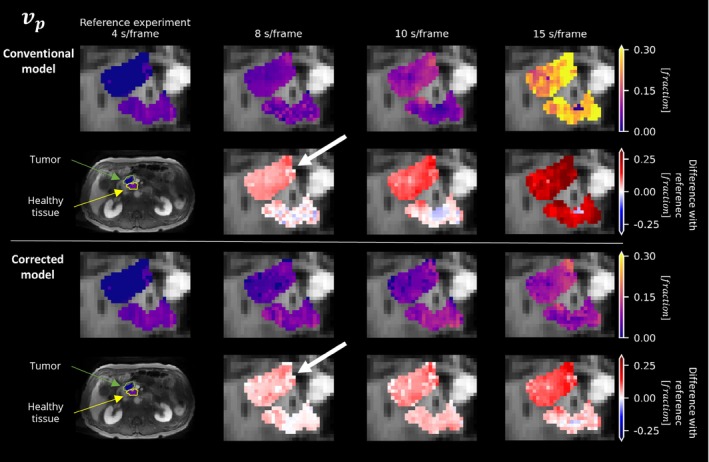
Estimation of the parameter $v_{p}$ in the tumor (*green arrow*) and healthy tissue (*yellow arrow*) of a pancreatic ductal adenocarcinoma patient. The results were calculated using both the conventional model (*Row 1*) and the corrected model (*Row 3*) at different temporal resolutions. Additionally, corresponding differences in predictions (*Rows 2 and 4*) across various temporal resolutions compared with the reference (*Column 1*), which correspond to the fastest acquisition, are shown.

**FIGURE 8:**
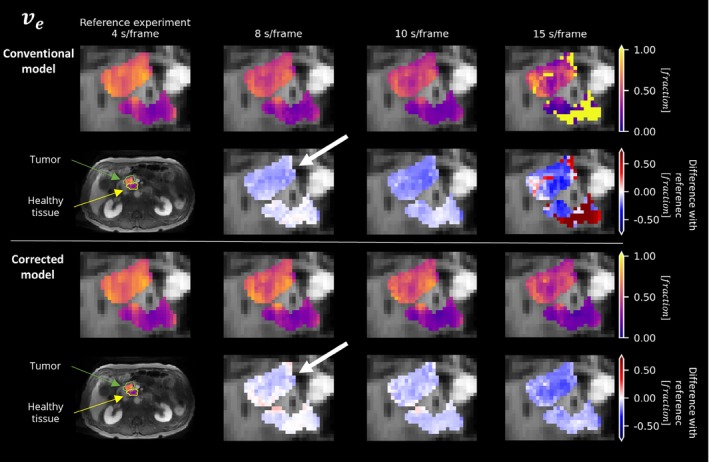
Estimation of the parameter $v_{e}$ in the tumor (*green arrow*) and healthy tissue (*yellow arrow*) of a pancreatic ductal adenocarcinoma patient. The results were calculated using both the conventional model (*Row 1*) and the corrected model (*Row 3*) at different temporal resolutions. Additionally, corresponding differences in predictions (*Rows 2 and 4*) across various temporal resolutions compared with the reference (*Column 1*), which correspond to the fastest acquisition, are shown.

**FIGURE 9:**
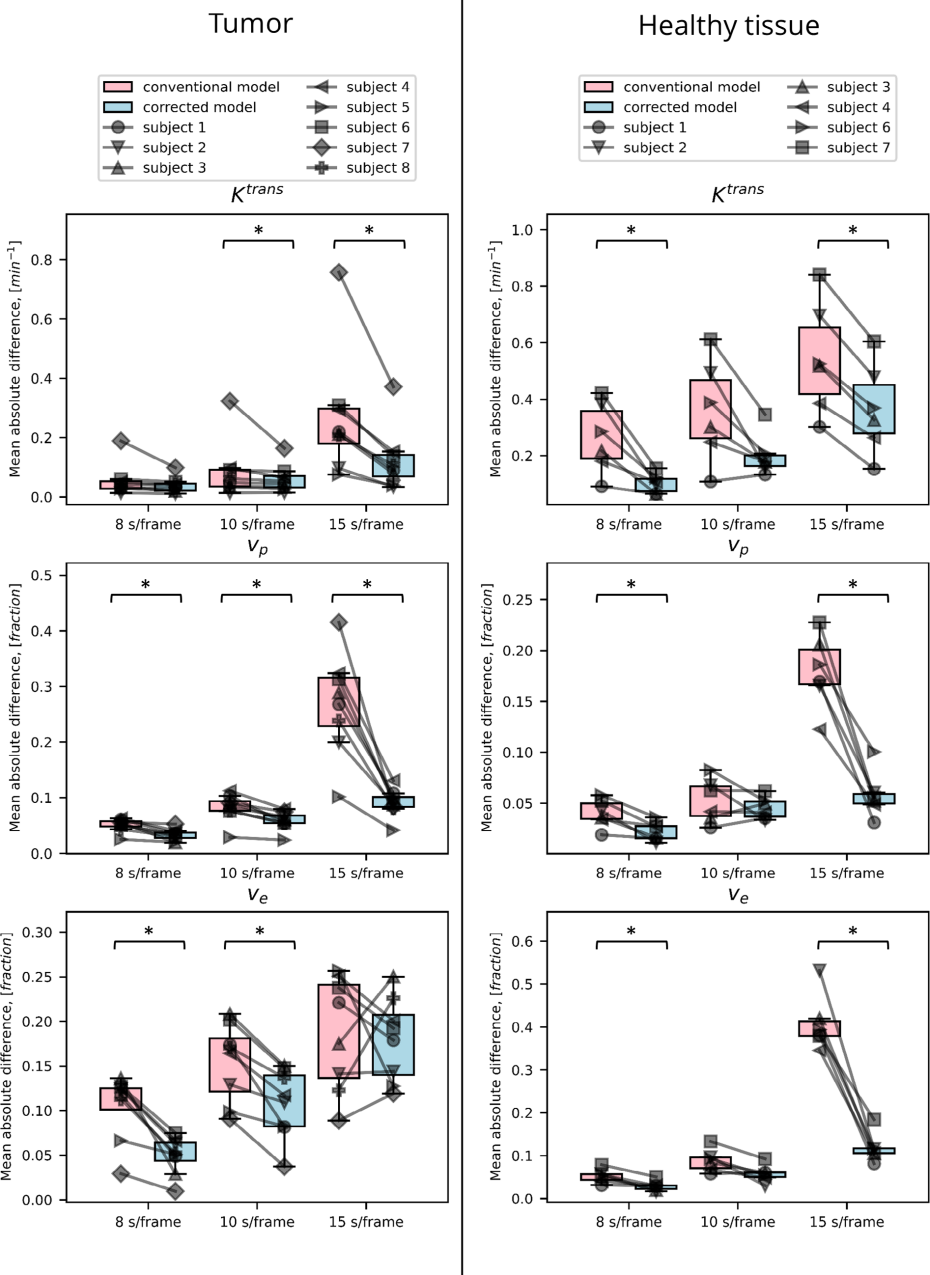
The mean absolute difference (MAD) of predicted parameters was obtained at various temporal resolutions compared with the reference acquisition, which had the fastest temporal resolution of 4 s/frame. MAD values are presented for both the tumor (*left*) and healthy tissue (*right*). A lower MAD value indicates a closer agreement between the predicted parameters and the reference, reflecting improved accuracy in parameter estimation.

Overall, the in vivo experiments (Figures 6, 7, 8, 9) provided empirical evidence supporting the effectiveness of the corrected extended Tofts model in accurately estimating pharmacokinetic parameters from averaged signals.

The corrected model demonstrated greater consistency over different temporal resolutions compared with the conventional model, suggesting its ability to handle averaging signals more effectively. Despite the small number of patients, the corrected model was significantly more consistent in its parameter estimates in 13 of 18 comparisons (3 parameters, 3 temporal resolutions, 2 ROIs). Moreover, at a temporal resolution of 4 s/frame, the parameter $v_{p}$ shows a clear distinction between tumor and healthy tissue, which is better preserved at worse temporal resolutions (>4 s/frame) when using the corrected model (Figure 8). A similar, although less pronounced, effect was observed in parameters $K^{\text{trans}}$ and $v_{e}$.

## 4 DISCUSSION AND CONCLUSIONS

This work demonstrates that modern acquisitions that oversample the center of k‐space introduce a systematic and random error in the parameter estimation if not properly considered in the modeling. Our correction of the extended Tofts model accounts for this and results in accurate and precise parameter estimates. In simulations, we showed that the proposed model outperforms conventional modeling for estimating all pharmacokinetic parameters, with the largest effect in $v_{p}$. Moreover, our simulations showed that, when properly considered, variable density sampling outperforms conventional discrete sampling, especially for lower temporal resolutions. This improved behavior can be attributed to the additional temporal information inherent in time‐averaged signals—a feature absent in discrete acquisition. Our in vivo findings support the conclusions drawn from the simulations. Our new model will help the introduction of novel acquisition techniques to perform accurate and precise DCE modeling. Moreover, it suggests that such new acquisition techniques, when properly modeled, may have unanticipated added benefits of better retaining fast (subtemporal resolution) processes, as compared with conventional acquisitions. This behavior was observed in simulations and in vivo, in which parameter maps remained more consistent between reconstruction times.

Building on this thought, it may be hypothesized that for variable‐density sampling trajectories, the temporal resolution becomes less important. Ironically, these acquisitions were partially developed to enable higher temporal resolutions. However, if temporal resolution becomes less crucial for accurate pharmacokinetic modeling, we could consider increasing spatial resolution, reducing acceleration factor, and/or including motion robustness (e.g., soft‐gating^27^, ^28^, ^29^ typically requires dynamic times that are longer than a respiratory cycle). Longer temporal frames may also loosen the need for temporal regularization; such regularization can affect the accuracy of pharmacokinetic modeling.^30^

Additionally, simulated experiments have revealed that the choice of AIF, temporal resolution, and the implementation of the extended Tofts model might influence the estimation of pharmacokinetic parameters. When using a patient‐specific AIF derived from a time‐averaged acquisition, Eq. (3) suggests that the correction of a pharmacokinetic model is unnecessary. However, Supporting Information S4 shows that this only works when taking care of the implementation. In our naïve implementation, discrepancy was caused by two factors: the first being a result of discrete sampling of the AIF, and the second being related to the choice of model implementation. Our implementation has an analytical description of the AIF, which was designed with non‐convolved AIF signals in mind. The analytical equation does not have the degrees of freedom to describe the convolved AIF accurately.

Another important point is that, in vivo, a fair comparison of approaches is challenging and would require administering two doses of contrast agent. With no direct patient benefit, this was considered unethical. Even if two doses are administered, the second DCE acquisition would be affected by the first, given that complete contrast‐agent elimination takes approximately 48 h. Hence, our only metric in vivo was model consistency. One challenge in this approach is that the “best” modeling is done on high temporal resolutions, which typically contain the most noise. This noise adds to additional uncertainty that may mask some of the benefits. That said, the conducted in vivo experiments agreed with the simulations and supported our equation.

In this work, we chose to build on the computationally convenient analytical representation of the extended Tofts model and managed to implement additional convolution analytically, too. This does come at the cost of working in the concentration domain. One might argue that temporal averaging happens in the signal space and that modeling the averaging in the concentration space might be affected by the nonlinear relation between signal and concentration. Note that in our approach, the global nonlinearity (e.g., nonlinear effect occurring between precontrast and max contrast) was corrected for by converting the dynamic signal to the concentration curve with the spoiled gradient‐echo signal model and considering the specific relaxivities of the paramagnetic agent. However, local nonlinear effects, occurring during the averaging of one dynamic, may still play a role. In Supporting information S5, following Quantitative Imaging Biomarkers Alliance–recommended guidelines,^31^ we show that these nonlinear effects do not significantly affect the accuracy when accounting for temporal averaging in concentration space. That said, an alternative approach may be to fit directly in the signal domain, where the time averaging is applied by numerical convolution of the signal function with a rectangular function, as follows:

(5) $$S^{\prime }\left(t\right)=\frac{1}{∆t}\text{rect}\left(\frac{t}{∆t}\right)*S\left(t\right)$$

where $S^{\prime }\left(t\right)$ is the convolved signal; ∆t is the measuring time per dynamic; and S(t) is the signal equation for data following the extended Tofts model (see Supporting information S5 for details). This numerical convolution can be made arbitrarily accurate by numerically sampling S(t) with small steps (e.g., we chose 0.1 s) and fitted to the actual signal. Although this equation better represents the imaging physics, it loses its analytical character. In Supporting information S5, we see that this approach reaches very comparable performance as convolving in the concentration domain.

Finally, in vivo DCE‐MRI signals typically exhibit Rician noise, which when converted to concentration space can be more complex. With the lack of a noise model in the concentration domain, and to avoid additional biases of Rician noise, we implemented Gaussian noise in our simulations. This choice allows us to focus on the models' accuracy without additional noise‐related biases skewing parameter estimations and obscuring the models' performances. Additionally, for SNR values above 3, Rician noise can be well‐approximated by Gaussian noise.^32^ In our simulations, the average SNR in the concentration domain was 20, and we do not expect Rician noise to cause a bias in that case. Although the SNR precontrast would be 0 in the contrast‐concentration domain, for most tissues it is nonzero in the signal domain (in which noise is Rician).

### 4.1 Limitations

One drawback is the limited amount of controlled experimental validation of the new equation. Unfortunately, we do not have an established DCE phantom that models the dynamic microvasculature behavior, and we had no ground‐truth in vivo references. However, the experiments that we performed do support the equation. First, the equations are derived using basic imaging concepts and hence should be correct. Second, Eq. (3) was validated using open source–tested software. Third, we validated it in vivo, where the model was consistent over sampling rates.

Another shortcoming lies in our use of compressed sensing—a technique designed for the reconstruction of undersampled k‐space. Its temporal regularization may introduce blurring to the temporal signal, resulting in some inaccuracy in our suggested equation. It would be interesting to explore the possibility of accounting for this effect by incorporating it as an additional blurring factor in Eq. (2) in future work. However, as we can now model longer dynamic scan times more accurately, potentially, temporal regularization is less necessary.

## 5 Conclusions

In conclusion, temporally averaged signals acquired with variable density trajectories oversampling the k‐space center require modification of pharmacokinetic modeling to obtain accurate and precise parameter estimates, especially at low temporal resolutions. Our proposed correction showed improved estimates of pharmacokinetic parameters in simulations and in vivo and can be directly adapted. The correction will help with the implementation of modern variable density acquisition techniques as a tool for DCE‐MRI.

## FUNDING INFORMATION

This work was funded by the KWF Dutch Cancer Society (KWF‐UVA 2021. 13785; O.G.‐C.) and the Cancer Center Amsterdam (CCA 2020‐7‐01; O.G.‐C. and N.K.). A modified version of the Amsterdam UMC “Prospective Undersampling in Multiple Dimensions” (PROUD) software patch was used for in vivo scans.

## CONFLICT OF INTEREST

Oliver J. Gurney‐Champion discloses a pending patent on a different DCE topic.

## Supporting information


**Equation S1.** Proof of Eq (3). Equation S2. Definition of analytical AIF proposed by Orton et al^16^. Equation S3. Definition of time‐averaged AIF. Figures S4. Results of repeated Experiments 1 and 2 with time‐averaged AIF. Figures S5. Comparison of time‐averaging implmentations in the concentration and signal domains.
