# Supplementary material for: A correction for modeling radial, spiral, and PROPELLER dynamic contrast‐enhanced data: Time‐averaged extended Tofts
Source: Magn Reson Med. 2025 Mar 30;94(2):810–24. doi: 10.1002/mrm.30514 (PMC12137790; doi:10.1002/mrm.30514)
Supplement: Supplementary file 1 — Equation S1. Proof of Eq (3). Equation S2. Definition of analytical AIF proposed by Orton et al16. Equation S3. Definition of time‐averaged AIF. Figures S4. Results of repeated Experiments 1 and 2 with time‐averaged AIF. Figures S5. Comparison of time‐averaging implmentations in the concentration and signal domains. [file MRM-94-810-s001.docx]

**Supporting information**

**A correction for modeling radial, spiral, and PROPELLER DCE data: time-averaged extended Tofts**

**Natalia V. Korobova, Nienke P. M. Wassenaar, Marian A. Troelstra, Eric M. Schrauben, Oliver J. Gurney-Champion**

**S1.**

$\begin{aligned} C^{'}\left( t,u,C_{p} \right)=\frac{1}{\Delta t}C\left( t,u,C_{p}\left( t \right) \right)*rect\left( \frac{t}{\Delta t} \right)=C\left( t,u,{\frac{1}{\Delta t}C}_{p}\left( t \right)*rect\left( \frac{t}{\Delta t} \right) \right)=C\left( t,u,C_{p}'\left( t \right) \right) \end{aligned}$ [S1.1]

If we consider:

$\begin{aligned} f=rect\left( \frac{t}{\Delta t} \right) \end{aligned}$[S1.2]

$\begin{aligned} g=C_{p}\left( t,\theta_{AIF} \right) \end{aligned}$ [S1.3]

$\begin{aligned} h=e^{-\frac{K^{trans}}{v_{e}} t} \end{aligned}$ [S1.4]

then,

$\begin{aligned} C^{'}\left( t,u,C_{p} \right)=\frac{1}{\Delta t}C\left( t,{u,C}_{p}\left( t \right) \right)*rect\left( \frac{t}{\Delta t} \right)= \\ \frac{1}{\Delta t}\left( v_{p}g \right)*f+\frac{1}{\Delta t}K^{trans}\left( g*h \right)*f= \\ v_{p}\left( \frac{1}{\Delta t}g*f \right)+K^{trans}\left( \frac{1}{\Delta t}g*f \right)*h= \\ C\left( t,u,{\frac{1}{\Delta t}C}_{p}\left( t \right)*rect\left( \frac{t}{\Delta t} \right) \right) \\ =C\left( t,u,C_{p}'\left( t \right) \right) \end{aligned}$ [S1.5]

**S2.**

The analytical arterial input function earlier proposed by M. R. Orton et al^15^:

$\begin{aligned} C_{p}\left( t,\theta_{AIF} \right)=\left\{ \begin{matrix} a_{B}\left( 1-\cos\left( \mu_{B}t \right) \right)+a_{B}a_{G}f\left( t,\mu_{G} \right) & for & 0\leq t\leq t_{B} \\ a_{B}a_{G}f\left( t_{B},\mu_{G} \right)e^{-\mu_{G}\left( t-t_{B} \right)} & for & t>t_{B} \end{matrix} \right., \end{aligned}$ [S2.1]

where $\theta_{AIF}=\{a_{B},a_{G},\mu_{B},\mu_{G}\}$, $t_{B}=2\pi\mu_{B}^{-1}$ and

$$f\left( t,\alpha\right)=\frac{1}{\alpha}\left( 1-e^{-\alpha t} \right)-\frac{1}{\alpha^{2}+\mu_{B}^{2}}\left( \alpha\cos\left( \mu_{B}t \right)+\mu_{B}\sin\left( \mu_{B}t \right)-\alpha e^{-\alpha t} \right).$$

Parameters $\theta_{AIF}=\{a_{B},a_{G},\mu_{B},\mu_{G}\}$ define the shape of arterial input function and are usually obtained by fitting this analytical model $C_{p}\left( t,\theta_{AIF} \right)$ to the DCE signal.

**S3.**

Using Eqs. 4 and S2.1, the analytical input function corrected for the time-averaging is

$$C_{p}^{'}\left( t,\theta_{AIF} \right)=\frac{a_{B}}{\Delta t}\left( f\left( t+\frac{\Delta t}{2},0 \right)-f\left( t-\frac{\Delta t}{2},0 \right) \right)+$$

$$+\frac{a_{B}a_{G}}{\Delta t\mu_{G}}\left( f\left( t+\frac{\Delta t}{2},0 \right)-f\left( t-\frac{\Delta t}{2},0 \right)-f\left( t+\frac{\Delta t}{2},\mu_{G} \right)+f\left( t-\frac{\Delta t}{2},\mu_{G} \right) \right)$$

for $0\leq t\leq t_{B}$, and it is

$$C_{p}^{'}\left( t,\theta_{AIF} \right)=\frac{a_{B}}{\Delta t}\left( f\left( t_{B}+\frac{\Delta t}{2},0 \right)-f\left( t_{B}-\frac{\Delta t}{2},0 \right) \right)+$$

$$\frac{a_{B}a_{G}}{\Delta t\mu_{G}}\left( \begin{aligned} f\left( t_{B}+\frac{\Delta t}{2},0 \right)-f\left( t_{B}-\frac{\Delta t}{2},0 \right)-f\left( t_{B}+\frac{\Delta t}{2},\mu_{G} \right)e^{-\mu_{G}\left( t+\frac{\Delta t}{2}-t_{B} \right)} \\ +f\left( t_{B}-\frac{\Delta t}{2},\mu_{G} \right)e^{-\mu_{G}(t-\frac{\Delta t}{2}-t_{B})} \end{aligned} \right)$$

for $t>t_{B}$.

The derivation of this equation relies on two key principles. First. the rectangular function in Eq. [4] can be represented as the sum of two Heaviside step functions, each shifted relative to the other along the temporal axis by $\Delta t$. Second, a Heaviside function can be expressed as an exponential function with the coefficient of zero.

**S4.**

Our intention in the following experiments was to put Eq. [3] into practical use and show that one does not need to consider the convolution if a patient-specific AIF is measured. Therefore, Experiments 1 and 2 were modified by using a temporally averaged (patient-specific) AIF $C_{p}^{'}$ instead of discretely sampled AIF. We conducted experiments by performing two fits: one with the conventional model (Experiment 1 with time-averaged AIF) and one with the corrected model (Experiment 2 with time-averaged AIF), both fitting a convolved model to the AIF as using a convolved model for the signal.

Figures S4.1-S4.3 present estimated parameters ${K^{trans}, v}_{p}, v_{e}$ obtained from fitting both models. Despite the initial expectation based on Eq. [3] that, with a patient-specific AIF $C_{p}^{'}$, the conventional and corrected models would yield similar results, our calculations showed the opposite: parameters estimated with the conventional model were less accurate and precise.

There were two factors contributing to a poor translation to practice. The first factor is that we were solving an inverse problem where parameters were fitted to discretely measured data. While the direct computation of Eq. [3] is straightforward, solving its inverse problem is more complex due to the discretization, the choice of sampling intervals, and the fitting process itself, all of which can introduce errors. The second factor is that in these experiments, we are using an analytical description of the AIF^15^, which was developed with a non-convolved signal in mind. The analytical equation does not have the degrees of freedom to describe the convolved AIF accurately.

An alternative implementation of the model such as LEK_UoEdinburgh from the OSIPI GitHub^16^, that does not parametrize the AIF could be the solution. However, in preliminary experiments (data not shown), this model still showed a discrepancy at low temporal resolutions. The numerical integration required for unparameterized AIF is less accurate at lower temporal resolutions. This leads to underestimation of the concentration curves and, consequently, a poor prediction of the pharmacokinetic parameters.

To further verify Eq. [3] as accurately as possible, we mimicked the view-sharing reconstruction approach, where the virtually acquired temporal resolution of 15 s/frame (to mimic temporal blurring) was much lower than the virtual temporal sampling of 0.1 s/frame (to ensure sufficient sampling). The simulated data $C^{'}\left( t,u,C_{p} \right)$ (with the equations from Supporting Information S3) represented the left side of Eq. [3], while the fitted model $C\left( t,u,C_{p}'\left( t,\theta_{AIF} \right) \right)$ represented the right side of Eq. [3]. Therefore, Eq. [3] was considered to be valid if the fit adequately described the data. To remove any assumptions on the shape of the AIF, we used the extended Tofts implementation from LEK_UoEdinburgh from the OSIPI GitHub^16^. Figure S4.4 shows the results of this experiment. From these findings, we established a good resemblance between predicted parameters and the ground truth, validating Eq. [3].


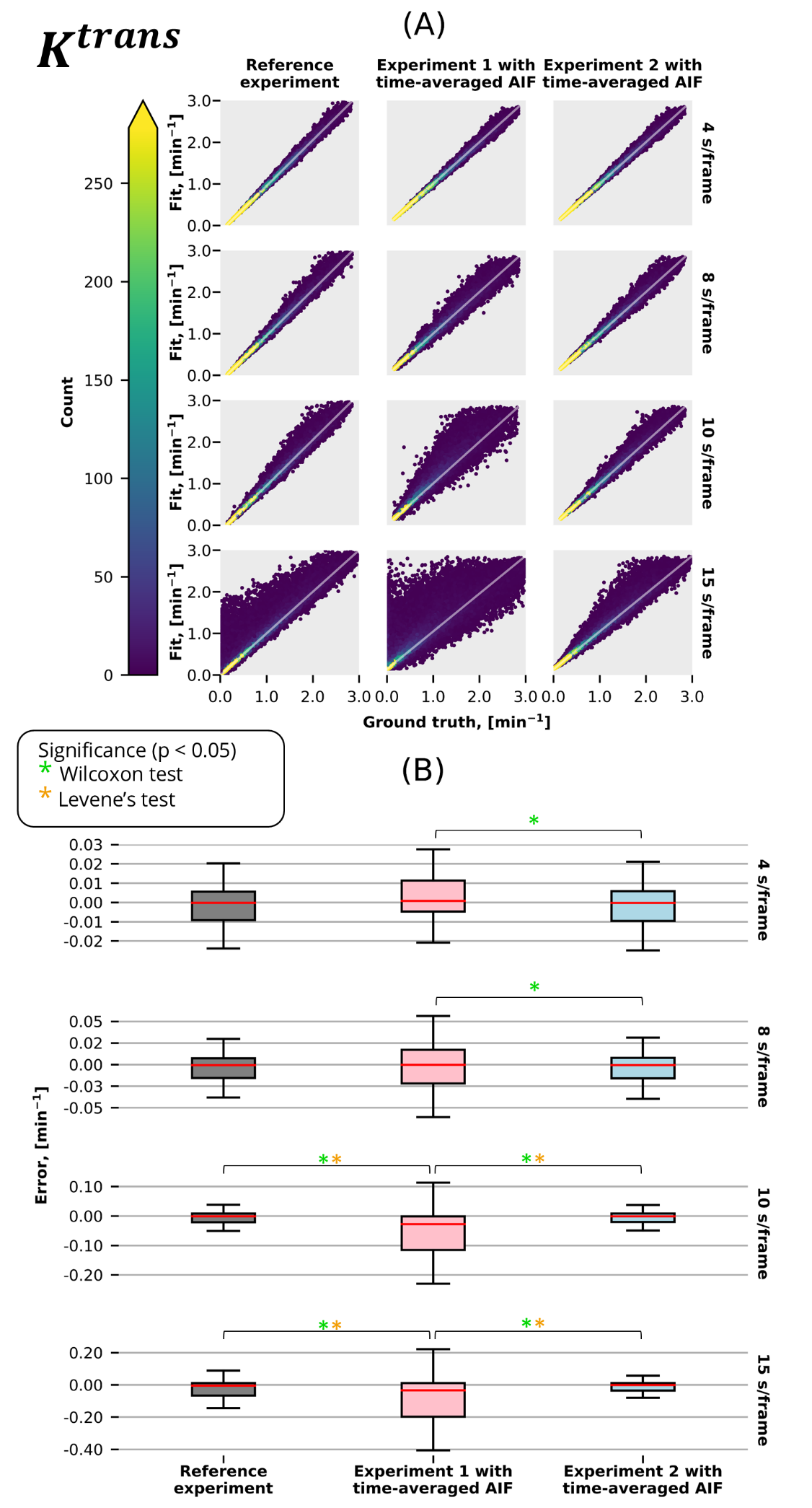


Figure S4.1. The $K^{trans}$ results of the experiments at various temporal resolutions. A. The graph displays the fitted value of the parameter $K^{trans}$ plotted against the ground truth. The colour gradient represents the density of points, while the white diagonal line represents the function Fit=Ground Truth. B. The distribution of errors in the predicted parameter $K^{trans}$ for the same experiments and temporal resolutions. Significant differences, as identified by the Wilcoxon (accuracy) and Lavene (precision) tests (p < 0.05), are denoted by green and orange stars, respectively.


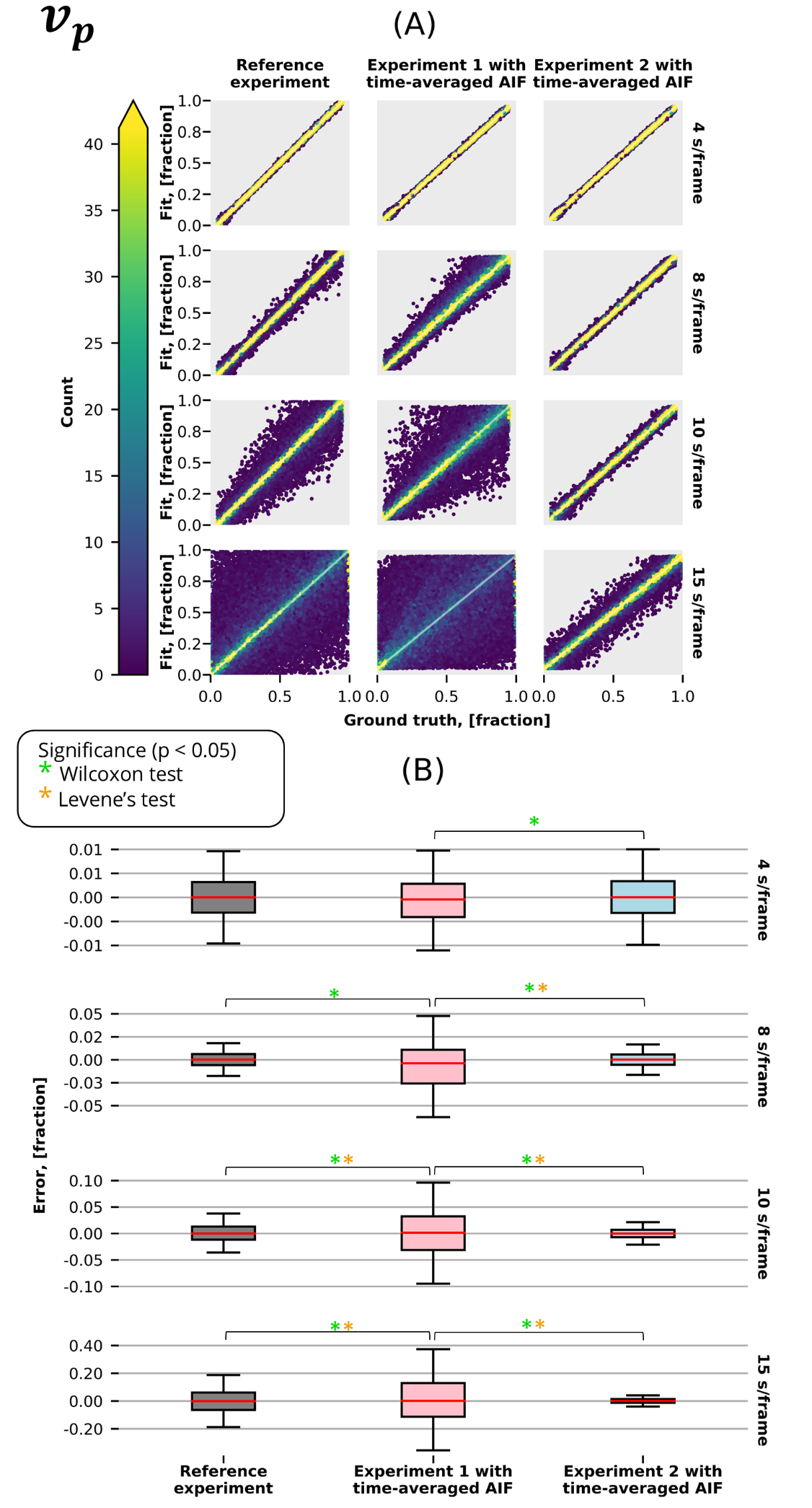


Figure S4.2. The $v_{p}$ results of the experiments at various temporal resolutions. A. The graph displays the fitted value of the parameter $v_{p}$ plotted against the ground truth. The colour gradient represents the density of points, while the white diagonal line represents the function Fit=Ground Truth. B. The distribution of errors in the predicted parameter $v_{p}$ for the same experiments and temporal resolutions. Significant differences, as identified by the Wilcoxon (accuracy) and Lavene (precision) tests (p < 0.05), are denoted by green and orange stars, respectively.


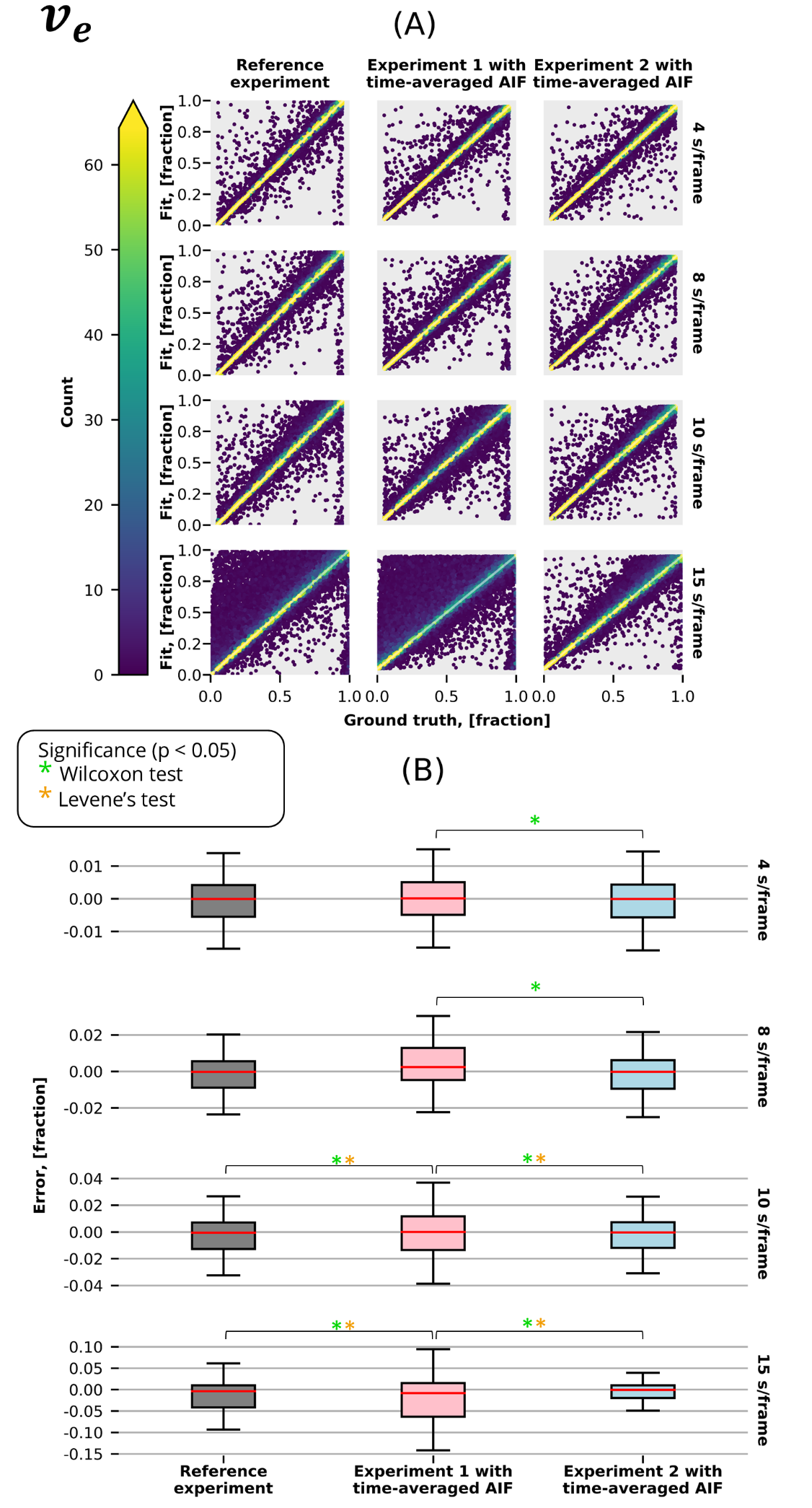


Figure S4.3. The $v_{e}$ results of the experiments at various temporal resolutions. A. The graph displays the fitted value of the parameter $v_{e}$ plotted against the ground truth. The colour gradient represents the density of points, while the white diagonal line represents the function Fit=Ground Truth. B. The distribution of errors in the predicted parameter $v_{e}$ for the same experiments and temporal resolutions. Significant differences, as identified by the Wilcoxon (accuracy) and Lavene (precision) tests (p < 0.05), are denoted by green and orange stars, respectively.


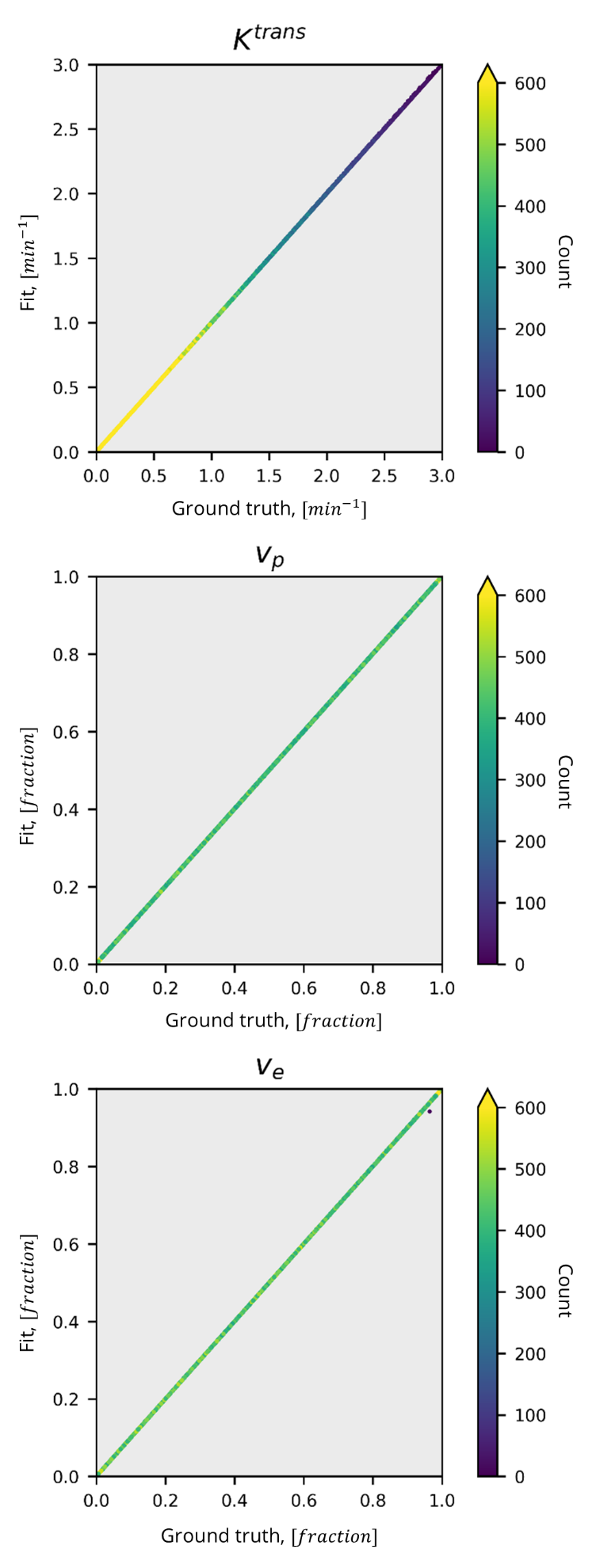


Figure S4.4.The results of the experiment validating Equation 3. The graphs display the fitted value of the parameters $K^{trans}$, $v_{p}$, and $v_{e}$ plotted against the ground truth. The color gradient represents the density of points at each location.

**S5.**

To investigate the potential impact of the non-linearities in the signal-concentration dependency, we performed additional simulations. Generally, a linear relation between signal and concentration happens either at larger flip angles or low values of concentration. According to the most recent recommendations from the Quantitative Imaging Biomarker Alliance (QIBA), the recommended flip angle for DCE imaging at 3T ranges between 10-15° for brain and prostate imaging and 10-30° for breast imaging. In alignment with these guidelines, our in vivo data for pancreas imaging was acquired at a flip angle of 25°. This is at the high-end of the flip angle ranges and hence if there is no impact of non-linearity here, one can conclude it is safe to work in the concentration domain.

We examined whether the implementation of time-averaging fit in concentration space results in less accurate and/or precise estimates due to local non-linearities. To test this, we simulated 50,000 signal curves for flip angles of 10°, 15°, and 25° (with $T_{1}$ =725 ms, corresponding to pancreas tissue). To achieve this, we first generated concentration curves $C\left( t,u,C_{p} \right)$ every 0.1 seconds using the conventional extended Tofts model and converted these to the effective relaxation time $T_{1}(t,u,C_{p},T_{10})$:

$$T_{1}\left( t,u,C_{p},T_{10} \right)=\frac{1}{r C\left( t,u,C_{p} \right)+\frac{1}{T_{10}}}$$

[S5.1]

where $r$ $[L/(mmol s)]$ is the relaxivity of the MR contrast agent, and $T_{10}$ is the baseline $T_{1}$ value. We then used a signal equation to calculate signal curves. Although, in general, the signal equation depends on the DCE acquisition, here we used the fast gradient echo equation:

$${S\left( t,u,C_{p},T_{10},M_{0},\alpha,TR \right)=M}_{0}\frac{\sin\alpha(1-e^{-\frac{TR}{T_{1}\left( t,u,C_{p},T_{10} \right)}})}{1- \cos\alpha e^{-\frac{TR}{T_{1}\left( t,u,C_{p},T_{10} \right)}}}$$

[S5.2]

where $M_{0}$ corresponds to spin density, $\alpha$ is the flip angle, and $TR$ is the repetition time. Note that when fitting $u$ to the signal curve, an additional parameter $M_{0}$ must also be fitted.

We subsequently performed signal averaging $S'\left( t,u,C_{p},T_{10},M_{0},\alpha,TR \right)$ by convolving it with a rectangular function:

$$S'\left( t,u,C_{p},T_{10},M_{0},\alpha,TR \right)=\frac{1}{\Delta t}rect\left( \frac{t}{\Delta t} \right)*S\left( t,u,C_{p},T_{10},M_{0},\alpha,TR \right).$$

[S5.3]

Using this approach, we simulated different temporal resolutions consistent with those used in the manuscript: 4, 8, 10, and 15 s/frame. Additionally, to ensure accurate estimation of $M_{0}$ from the fitting, we prolonged the pre-contrast phase to 10 acquisitions (0.7 - 2.5 min). This was especially important for the 15 s/frame acquisition. In practice, longer acquisition times in vivo will have higher SNR, reducing the potential issues with $M_{0}$ estimation. We added noise to the signal curves that corresponded to an average SNR of 60 in the signal domain (note noise levels in the manuscript were in the contrast concentration domain).

We performed fitting using three models:

1. Conventional fitting in the signal domain $S$.
2. Time-averaged fitting in the signal domain $S^{'}$.
3. Time-averaged fitting in the concentration domain $C^{'}$.
   - Here, concentration curves were calculated from the simulated signal curves prior to the fit by inverting of Eqs. S5.1-S5.2.

We compared these three fit approaches by analyzing their resulting error distributions.

The results (Figures S5.1-S5.3) clearly demonstrate that time-averaging fitting in the signal domain and time-averaging in concentration domain perform similarly across all three flip angles. Furthermore, both time-averaged models produced more accurate and more precise pharmacokinetic parameters than the conventional model across all flip angles. The low flip angle (10°) did result in similar performance between conventional and time-averaged models at lower temporal resolutions (up to 10 s/frame). This could be a result of overall lower sensitivity due to signal saturation for low flip angles masking additional effect of averaging.


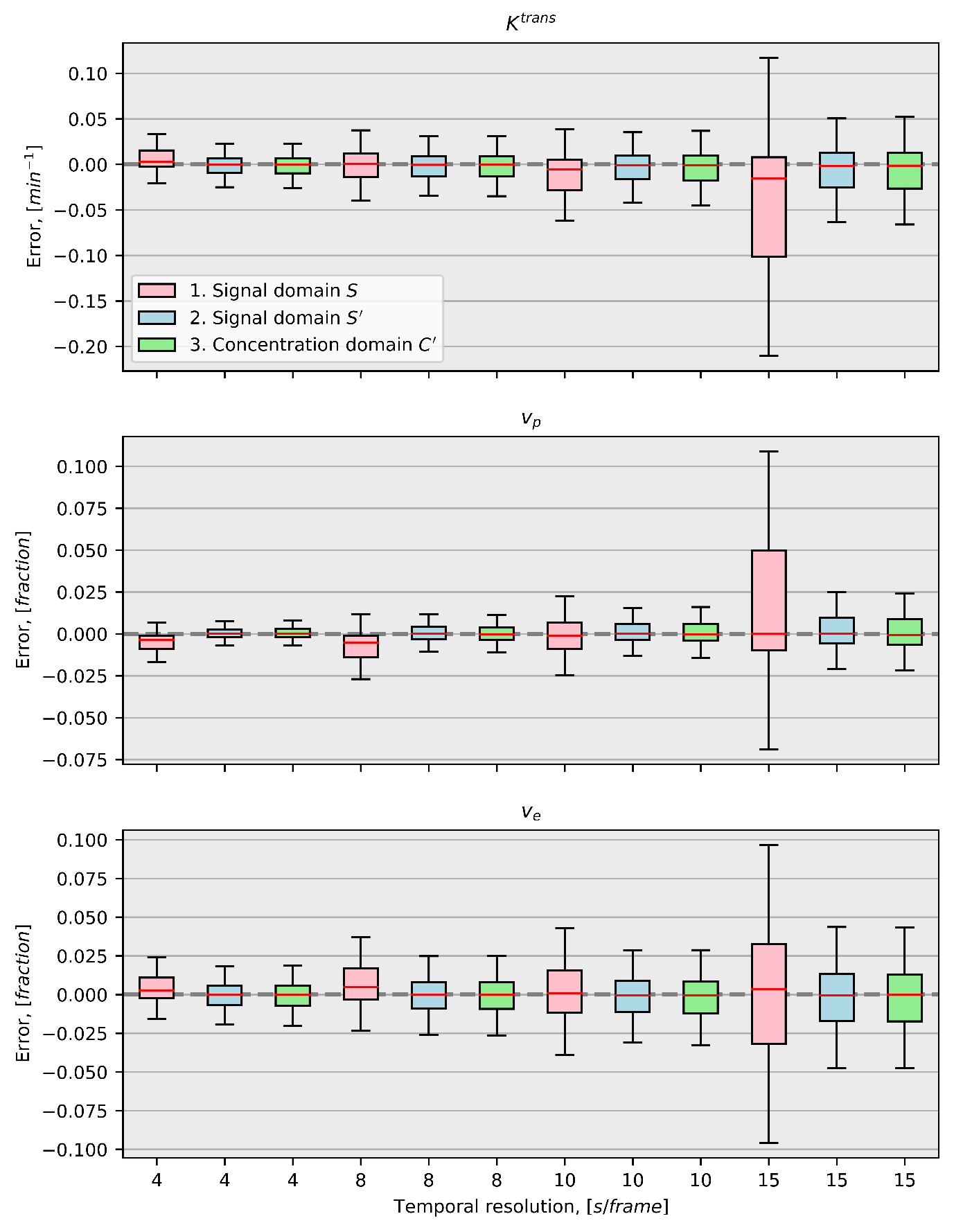


Figure S5.1. Error in the estimation of parameters $K^{trans}$ (top), $v_{p}$ (middle), $v_{e}$ (bottom) with the conventional fitting in the signal domain (pink), with the time-averaged fitting in the signal domain (blue), and with the time-averaged fitting in the concentration domain. The simulated signal was calculated with the **flip angle of 25˚** and temporal resolutions of 4, 8, 10, and 15 s/frame.


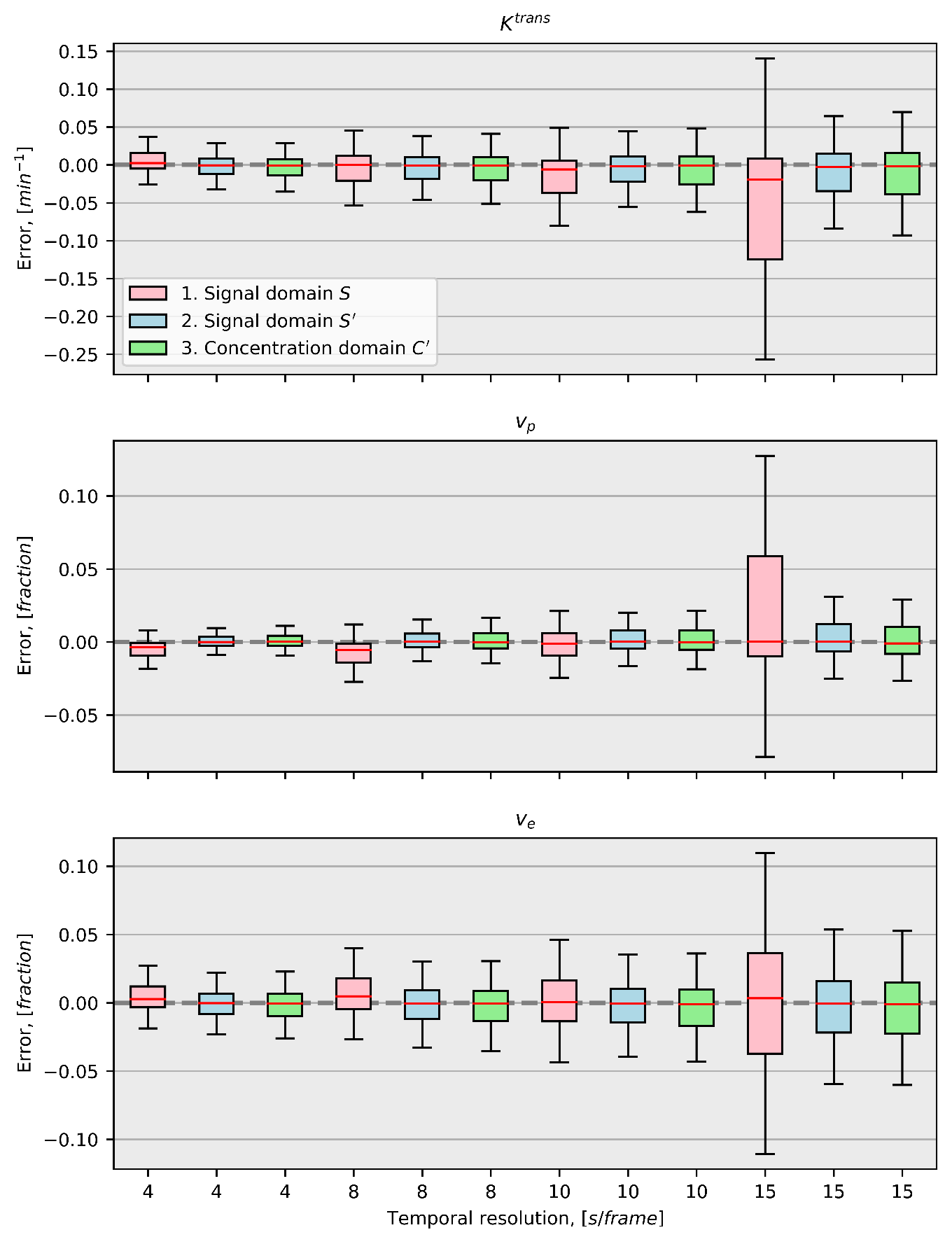


Figure S5.2. Error in the estimation of parameters $K^{trans}$ (top), $v_{p}$ (middle), $v_{e}$ (bottom) with the conventional fitting in the signal domain (pink), with the time-averaged fitting in the signal domain (blue), and with the time-averaged fitting in the concentration domain. The simulated signal was calculated with the **flip angle of 15˚** and temporal resolutions of 4, 8, 10, and 15 s/frame.


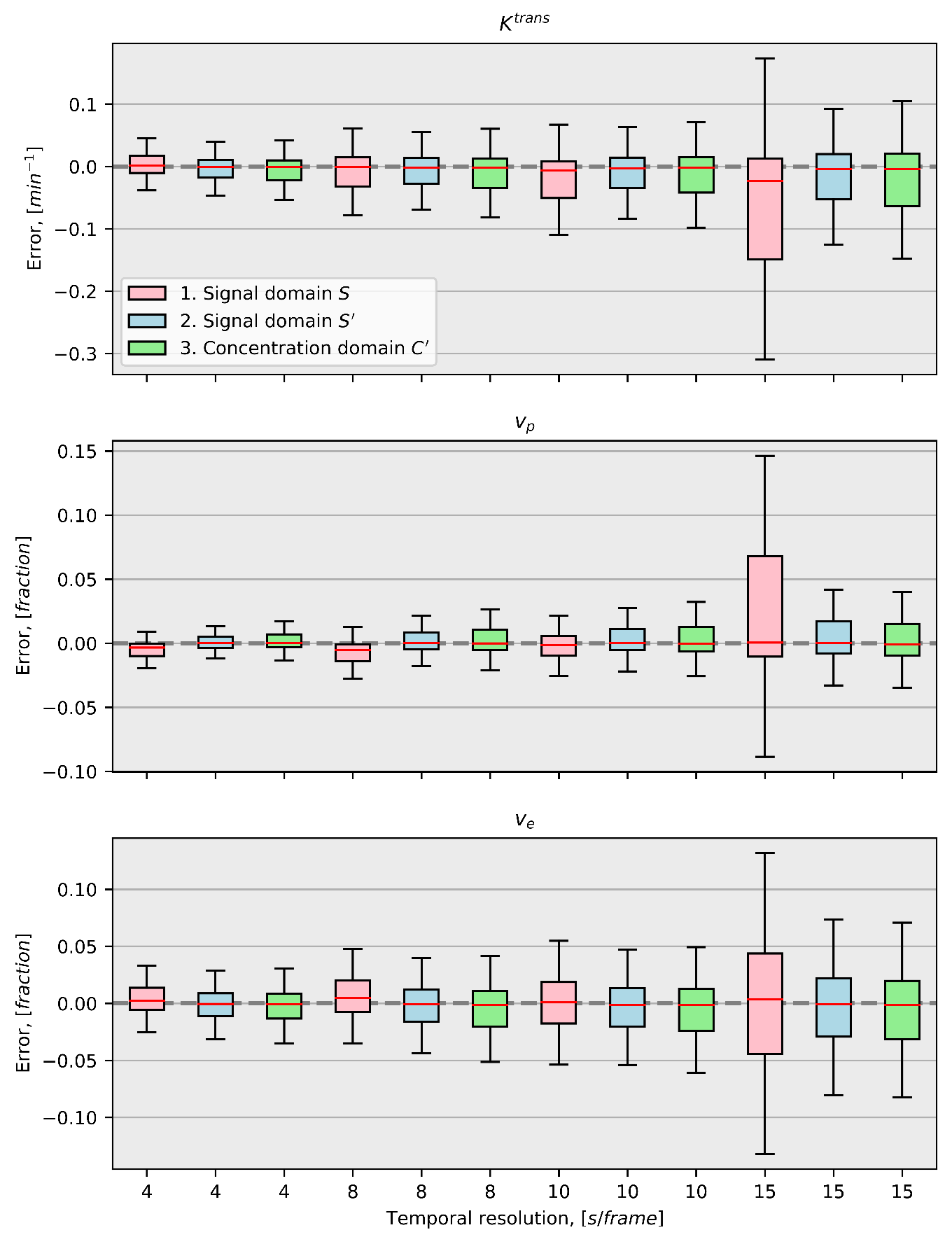


Figure S5.3. Error in the estimation of parameters $K^{trans}$ (top), $v_{p}$ (middle), $v_{e}$ (bottom) with the conventional fitting in the signal domain (pink), with the time-averaged fitting in the signal domain (blue), and with the time-averaged fitting in the concentration domain. The simulated signal was calculated with the **flip angle of 10˚** and temporal resolutions of 4, 8, 10, and 15 s/frame.
